# Optimizing Preparative Regimen for Umbilical Cord Blood Transplantation in Adult Acute Leukemia Patients: Acute Lymphoblastic Leukemia Requires Myeloablative Conditioning but Not Acute Myeloid Leukemia

**DOI:** 10.3390/jcm9072310

**Published:** 2020-07-21

**Authors:** Ja Min Byun, Junshik Hong, Doyeun Oh, Ho-Young Yhim, Young Rok Do, Joon Seong Park, Chul Won Jung, Deok-Hwan Yang, Jong-Ho Won, Hong Ghi Lee, Joon Ho Moon, Yeung-Chul Mun, Deog-Yeon Jo, Jae Joon Han, Je-Hwan Lee, Jae Hoon Lee, Junglim Lee, Sung-Soo Yoon

**Affiliations:** 1 Department of Internal Medicine, Seoul National University College of Medicine, Seoul National University Hospital, 101, Daehak-ro, Jongro-gu, Seoul 03080, Korea; jaminbyun@naver.com (J.M.B.); alertjun@hanmail.net (J.H.); 2 Department of Internal Medicine, CHA University School of Medicine, Seongnam 13496, Korea; doh@cha.ac.kr; 3 Department of Internal Medicine, Jeonbuk National University Medical School, Jeonju 54907, Korea; yhimhy@naver.com; 4 Department of Internal Medicine, Dongsan Medical Center, Keimyung University School of Medicine, Daegu 42601, Korea; dyr1160@dsmc.or.kr; 5 Department of Hematology-Oncology, Ajou University School of Medicine, Suwon 16499, Korea; jspark65@ajou.ac.kr; 6 Department of Internal Medicine, Samsung Medical Center, Sungkyunkwan University School of Medicine, Seoul 06351, Korea; chulwon1.jung@samsung.com; 7 Division of Hematology-Oncology, Chonnam National University Hwasun Hospital, Hwasun 58128, Korea; drydh1685@hotmail.com; 8 Division of Hematology and Medical Oncology, Department of Internal Medicine, Soonchunhyang University Seoul Hospital, Seoul 04401, Korea; jhwon.sch@gmail.com; 9 Division of Hematology-Oncology, Department of Internal Medicine, Konkuk University Medical Center, Seoul 05030, Korea; mlee@kuh.ac.kr; 10 Department of Hematology and Oncology, Kyungpook National University Hospital, School of Medicine, Kyungpook National University, Daegu 41944, Korea; jhmoon74@naver.com; 11 Department of Internal Medicine, Ewha Womans University School of Medicine, Seoul 07985, Korea; yeungchul@ewha.ac.kr; 12 Department of Internal Medicine, Chungnam National University College of Medicine, Daejeon 35015, Korea; deogyeon@cnu.ac.kr; 13 Department of Hematology and Medical Oncology, College of Medicine, Kyung Hee University, Seoul 02447, Korea; jchriste@naver.com; 14 Department of Hematology, Asan Medical Center, University of Ulsan College of Medicine, Seoul 05505, Korea; jhlee3@amc.seoul.kr; 15 Division of Hematology, Department of Internal Medicine, Gachon University College of Medicine Gil Medical Center, Incheon 21565, Korea; jhlee@gilhospital.com; 16 Division of Hematology and Medical Oncology, Department of Internal Medicine, Daegu Fatima Hospital, 99, Ayang-ro, Dong-gu, Daegu 41199, Korea

**Keywords:** cord blood transplantation, acute myeloid leukemia, acute lymphoblastic leukemia, conditioning

## Abstract

Cord blood transplantation (CBT) is a valuable alternative to bone marrow transplantation in adults without readily available donors. We conducted this study to investigate the feasibility of CBT for adult patients with acute leukemia with regards to impact of different conditioning and graft-versus-host disease (GVHD) prophylaxis regimens on clinical outcomes. From 16 centers in Korea, 41 acute myeloid leukemia (AML) and 29 ALL (acute lymphoblastic leukemia) patients undergoing CBT were enrolled. For AML patients, the neutrophil engraftment was observed in 87.5% of reduced intensity conditioning (RIC) and 72.0% of myeloablative conditioning (MAC) (*p* = 0.242). The median RFS was 5 months and OS 7 months. Conditioning regimen did not affect relapse free survival (RFS) or overall survival (OS). GVHD prophylaxis using calcineurin inhibitors (CNI) plus methotrexate was associated with better RFS compared to CNI plus ATG (*p* = 0.032). For ALL patients, neutrophil engraftment was observed in 55.6% of RIC and 90.0% of MAC (*p* = 0.034). The median RFS was 5 months and OS 19 months. MAC regimens, especially total body irradiation (TBI)-based regimen, were associated with both longer RFS and OS compared to other conditioning regimens. In conclusion, individualized conditioning regimens will add value in terms of enhancing safety and efficacy of CBT.

## 1. Introduction 

Allogeneic hematopoietic stem cell transplantation (HSCT) is the most effective anti-leukemic therapy for adults with acute leukemia. However, only about 30% of the patients have a human leukocyte antigen (HLA) identical sibling and additional 40%–50% matched unrelated donors; thus, many require an alternative donor. Two major alternative donor sources include haplo-identical donors and cord blood transplantation (CBT). Recent studies have established CBT as a valuable alternative to bone marrow transplantation in adults without readily available donors [1,2,3,4]. The immediate availability and the more leniently tolerated levels of HLA disparity in this setting, along with encouraging results showing better graft-versus-leukemia effects of CBT compared to HLA-matched or -mismatched unrelated donors [5,6,7,8], offer great advantages. On the other hand, the limited number of total nucleated cells (TNC) and low immunogenicity of the cells has been associated with slower hematopoietic and immune reconstitution [9,10]. Furthermore, the lack of prospective trial results raises contentions over optimal conditioning regimen and graft-versus-host disease (GVHD) prophylaxis during CBT. 

Recognizing the paucity of data on CBT from Korea, we conducted this retrospective study to investigate the feasibility of CBT for adult patients with acute leukemia with special regards to the impact of different conditioning and GVHD prophylaxis regimens on clinical outcomes. 

## 2. Materials and Methods 

### 2.1. Study Design and Subjects 

**Figure 1:**
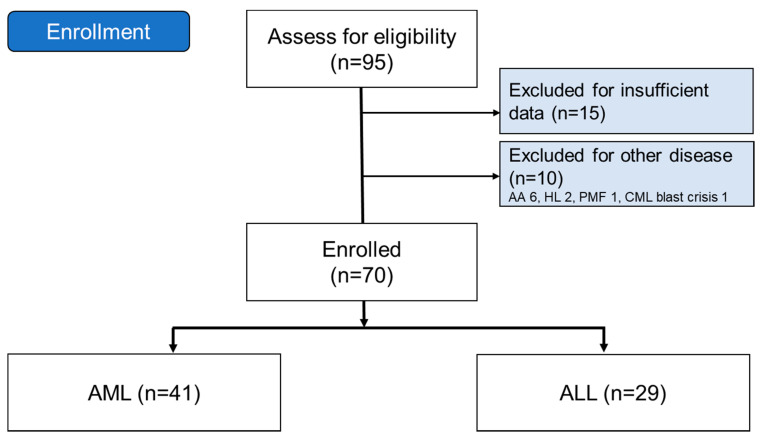
CONSORT diagram.

This was a multicenter, longitudinal cohort study of patients over 18 years old undergoing CBT. The study period was set between January 2001 and May 2019. A total of 95 patients were screened, and after elimination as shown in Figure 1, a total of 70 patients with acute myeloid leukemia (AML) and acute lymphoblastic leukemia (ALL) were deemed eligible (Figure 1). Their medical records were reviewed for demographics, response to treatment, factors related to CBT, and survival outcomes. This study was conducted according to the Declaration of Helsinki and was approved by the Institutional Review Board of each of the 16 participating hospitals. All authors had access to the study data and reviewed and approved this study.

### 2.2. HLA Typing and Donor Selection 

Donor-recipient matching considered lower resolution HLA matching at HLA-A and –B (antigen-level) and allele-level at HLA-DRB1. When double units were used, the transplant was assigned the lowest unit-recipient HLA-mismatch. In Korea, transplantation with CB units of 0 to 2 HLA antigen mismatches with the patient is recommended, but per physician’s decision 3/6 HLA matched CB unit can be used. Furthermore, the recommended minimum number of total nucleated cells (TNC) is 3.0 × 10^7^/kg of patient body weight at cryopreservation for malignancies. ABO incompatibility was not incorporated as one of the factors used in CB unit selection. 

### 2.3. Conditioning Regimen and GVHD Prophylaxis 

The conditioning regimens were divided into 4 groups: Group 1, reduced intensity conditioning (RIC) without total body irradiation (TBI); Group 2, RIC with TBI; Group 3, myeloablative conditioning (MAC) without TBI; Group 4, MAC with TBI. The conditioning regimens used for Group 1 (*n* = 14) consisted of the following: fludarabine plus busulfan (FluBu, *n* = 12) administered over 2 to 3 days, or, fludarabine plus melphalan (FluMel, *n* = 2). The regimen for Group 2 (*n* = 11) was fludarabine plus cyclophosphamide (FluCy) with TBI. The regimens used for Group 3 (*n* = 13) consisted of busulfan plus cyclophosphamide (BuCy, *n* = 3), thiotepa plus FluBu administered over 4 days (*n* = 3), and busulfan/melphalan/fludarabine (BuFluMel, *n* = 7). Finally, the regimen for Group 4 (*n* = 31) included TBI/cyclophosphamide plus fludararbine, etoposide or cytarabine. 

GVHD prophylaxis regimens included cyclosporine (CsA), methotrexate (MTX), mycophenolate mofetil (MMF), tacrolimus, and antithymocyte globulin (ATG). Tapering of immunosuppressants was initiated at 2–4 months after transplantation, with the goal of cessation by approximately 6 months in the absence of GVHD. Supportive care after transplantation, including empirical antibiotics use, cytomegalovirus (CMV) prophylaxis and hepatic sinusoidal obstruction syndrome (SOS) prevention, was performed according to each institutional protocol. 

### 2.4. Definitions 

Neutrophil engraftment was defined as an absolute neutrophil count (ANC) > 0.5 × 10^9^/L for 3 consecutive days. Platelet recovery was defined as platelet count > 20.0 × 10^9^/L for 7 consecutive days without transfusion. Acute GVHD grading was performed according to the standard criteria [11]. Chronic GVHD was classified as mild, moderate, or severe according to the 2014 National Institutes of Health consensus criteria [12]. Treatment-related mortality (TRM) was defined as death without progression of underlying acute leukemia. Relapse was defined by the morphologic evidence of disease in the peripheral blood, bone marrow, or extra-medullary sites. The relapse free survival (RFS) was defined as the time from stem cell infusion to relapse or death from any cause. The overall survival (OS) was defined as the time from stem cell infusion to death of any cause. 

### 2.5. Statistical Analysis

Differences between groups were assessed using a Student’s t-test or one-way analysis of variance for continuous variables, and Pearson chi-square test for categorical variables, as indicated. The RFS and OS curves were estimated using the Kaplan–Meier method. If patients survived without death or progression, the survival was censored at the latest date of follow-up when no death or progression was confirmed. These data were analyzed using the Statistical Package for the Social Sciences software (IBM^®^ SPSS^®^ Statistics, version 22.0); *p*-values of <0.05 were considered statistically significant.

Cumulative incidence curves were used in competing-risk setting to calculate the probability of neutrophil and platelet engraftment, acute and chronic GVHD, and TRM. For neutrophil and platelet engraftment, death before recovery was considered as the competing events. For GVHD, death without an event was considered as the competing event. For TRM, relapse was considered as the competing event. For this part of the analyses, SAS Enterprise Guide 6.1 Version and the statistical software R (www.r-project.org) were used. Associations between potential prognostic factors and survival outcomes were evaluated using the Cox’s proportional hazard regression models. The following variables were considered as covariates: age at CBT, year of CBT, body weight, duration from diagnosis to CBT, HLA-mismatch, infused TNC and CD34+ cell dose, status of underlying disease at CBT, conditioning regimens, and GVHD prophylaxis. A stepwise backward procedure was used, and predictors achieving a *p*-value below 0.10 were considered then sequentially removed if the *p*-value in the multiple model was above 0.05.

## 3. Results

### 3.1. Patients 

The baseline characteristics of 41 AML and 29 ALL patients are shown in Table 1. The number of CBT cases increased over time for both AML and ALL. The median age at CBT was 47 years (range 22–66) for AML patients and 29 years (range 18–64) for ALL patients. The median time from diagnosis to transplantation was 7 months (range 2–85), and the median TNC was 2.5 × 10^7^/kg (range 1.0–5.2) and the median CD34 + cells 1.0 × 10^5^/kg (range 0.1–4.1).

### 3.2. Engraftment 

Fifty-five patients (78.6%) out of 70 achieved primary neutrophil engraftment at a median of day 22 (range 8–74) (Table 2). The cumulative incidence of neutrophil engraftment was 73.7% at day 22. Among the 15 patients who did not achieve neutrophil engraftment, 12 died and 3 survived without neutrophil engraftment. Generally, there was no difference between RIC conditioning versus MAC conditioning with regards to neutrophil engraftment (RIC 76% vs. MAC 80%, *p* = 0.696). More specifically, for AML patients the neutrophil engraftment was observed in 87.5% of RIC and 72.0% of MAC group (*p* = 0.242). For ALL patients, neutrophil engraftment was observed in 55.6% of RIC and 90.0% of MAC group (*p* = 0.034). The median time to platelet recovery was 46 days (range 15–182). The cumulative incidence of platelet engraftment was 88.2% by day 46. There was no difference in platelet recovery rates with regards to acute leukemia subtype or conditioning intensity.

### 3.3. GVHD 

As shown in Table 2, the cumulative incidence of any acute GVHD at day 100 for the entire cohort was 33.5%, and the cumulative incidence of grades II-IV acute GVHD was 24.7%. For AML patients, the cumulative incidence of any acute GVHD at day 100 was 29.3% and grades II-IV 26.8%. The cumulative incidence of severe acute GVHD (grades III and IV) was 12.2%. For ALL patients, the cumulative incidence of any acute GVHD at day 100 was 39.9% and grades II-IV 21.7%. The cumulative incidence of severe acute GVHD was 3.6%. 

The cumulative incidence of any chronic GVHD at 1 year was 17.8%, and the cumulative incidence of moderate to severe chronic GVHD was 3.2%. ALL patients showed trends towards more frequent incidence of chronic GVHD compared to AML patients (Table 2) but the difference did not reach statistical difference (for any chronic GVHD, *p* = 0.218; for moderate to severe chronic GVHD, *p* = 0.677). 

### 3.4. Complications other than GVHD 

At day 100, infections were documented in 45 patients (64.3%). They included bacterial (*n* = 34, 75.6%), fungal (*n* = 6, 13.3%), and viral infection (*n* = 5, 11.1%). Infection rates were not different (*P* = 0.305) between ATG users (55.0%, *n* = 11/20) versus ATG non-users (68.0%, *n* = 34/50). CMV antigenemia was detected in 45 patients (65.2%). Among them, 12 AML patients and 2 ALL patients had overt CMV disease. SOS occurred in three patients: one ALL patient who was conditioned with TBI/Cy died from complications related to SOS, while two AML patients (one undergoing TBI/Cy + cytarabine conditioning, and the other Bu/Flu/Mel) recovered. 

The cumulative incidence of TRM at Day 100 was 29.2%, and at year 1, 36.9%. A total of 28 patients died from TRM at a median of 70 days (range 9–1110). Causes of death included infection (*n* = 23), SOS (*n* = 1), heart failure (*n* = 2), acute respiratory distress syndrome (*n* = 1), and post-transplant lymphoproliferative disease (*n* = 1).

### 3.5. Relapse free Survival and overall survival of AML 

**Figure 2:**
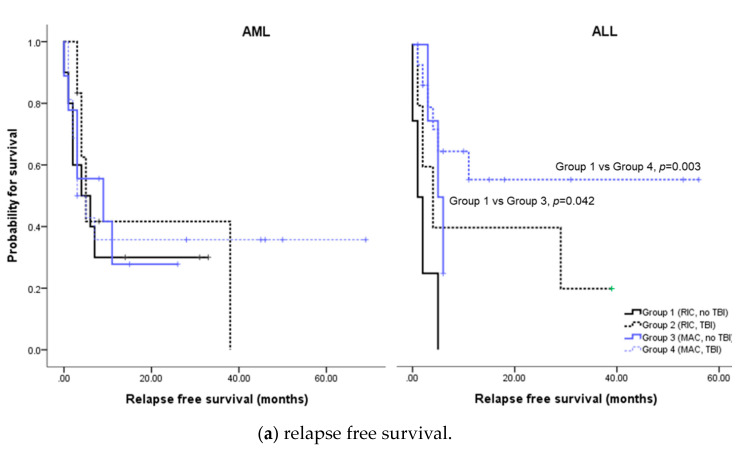

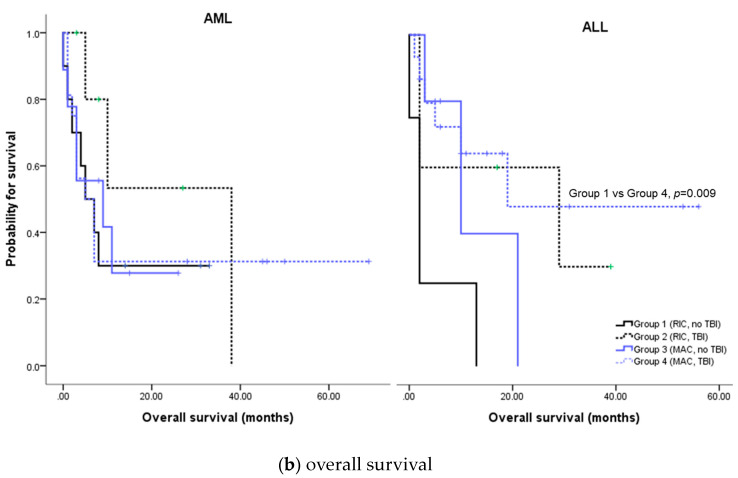
Survival according to conditioning regimen.

**Figure 3:**
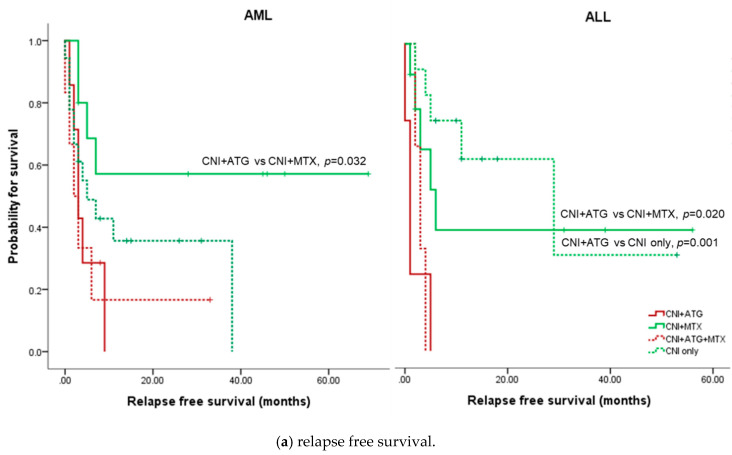

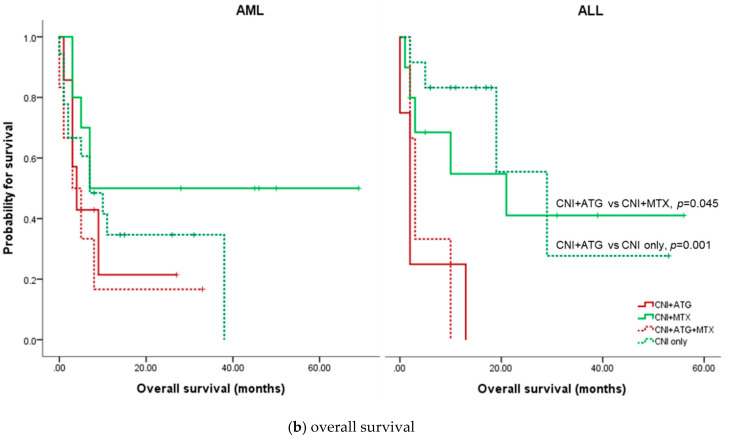
Survival according to graft-versus-host disease (GVHD) prophylaxis regimen.

The median RFS was 5 months and OS 7 months for AML patients. The 3-year RFS was 36.6% and 3-year OS 36.6%. Conditioning regimen did not affect RFS (Figure 2a) or OS (Figure 2b). GVHD prophylaxis using calcineurin inhibitors (CNI) plus MTX was associated with better RFS (median not reached) compared to CNI plus ATG (median 3 months, *p* = 0.032), as shown in Figure 3a. CNI plus MTX used also showed longest OS (Figure 3b) but the difference did not reach statistical significance. 

As shown in Table 3, multivariate analyses showed that the year of CBT and GVHD prophylaxis regimen were associated with RFS. Only the year of CBT was identified as prognostic factor for OS. The conditioning regimen did not affect survivals in AML. Double unit CB showed slightly better outcomes compared to single unit CB, but the difference was not statistically significant. 

### 3.6. Relapse Free Survival and Overall Survival of ALL

The median RFS was 5 months and OS 19 months for ALL patients. The 3-year RFS was 41.1% and 3-year OS 44.8%. Generally, MAC regimens were associated with better RFS (Figure 2a). Especially, TBI-based MAC regimen was associated with both longer RFS and OS compared to other conditioning regimens. As in AML, ATG use was associated with worse survival in ALL (Figure 3). GVHD prophylaxis with CNI only was associated with best survival outcomes. 

Multivariate analyses identified the year of CBT, conditioning regimen, GVHD, and prophylaxis regimen as prognostic factors for RFS. Meanwhile, only conditioning regimen was recognized as prognostic factors for OS (Table 3). 

## 4. Discussion 

With the nuclear family becoming the dominant family unit, the interest in alternative donor sources for HSCT is growing. The present study provides evidence that CB is a good alternative cell source for adult acute leukemia patients with low incidence of GVHD. Furthermore, we found that individualized preparative regimens can improve the outcomes of CBT. More specifically, we found that (1) in line with previous reports [13,14], ATG should be used with caution during CBT; (2) incorporation of MTX may be beneficial; and (3) the use of MAC conditioning, especially TBI-based, for ALL improves survival outcomes, while there was no difference between MAC and RIC conditioning regimens for AML. 

The AML patients in the present study tended to be older than the ALL patients, but considering that median age of AML onset is 68, our patients with median age of 47 (range 22–66) represent relatively fit patient population. Even so, MAC conditioning regimens did not necessarily yield better outcomes compared to RIC conditioning regimens (Figure 2). Due to concerns over engraftment issues, initial adult CBT were carried out with MAC conditioning. However, after the introduction of Minnesota group’s regimen [15,16,17], the use of RIC conditioning is increasing and, accordingly, 39% of our AML patients also underwent RIC conditioning. The neutrophil engraftment was noted in 78% of the AML patients at a median of 21 days. More importantly, there were no difference between patients undergoing RIC conditioning versus MAC conditioning. For AML patients undergoing RIC conditioning, the neutrophil engraftment rate was 87.5% at a median of 19.5 days, which is similar to previous studies reporting neutrophil recovery ranging from 76% to 85% [15,18]. For those undergoing MAC conditioning, neutrophil recovery was seen in 72% at a median of 21 days. Furthermore, the 3-year RFS for our AML patients was 36.6%, which is comparable to previous studies reporting 3-year RFS ranging from 28% to 38% [15,17,18,19], suggesting that myelo-ablation is not absolutely necessary and conditioning regimen can be chosen based on the condition of the recipient for AML. 

The cumulative incidence of grades II-IV acute GVHD at Day 100 was 26.8%, which is comparable to previous reports and significantly less than HSCT from other alternative sources [17,20]. Interestingly, the use of MTX was associated with better survival outcomes (Figure 3). Traditionally in the United States and Europe, the use of MTX have been avoided due to the concern of engraftment [21]. On the other hand, there is evidence supporting the use of MTX from Japan [22,23,24,25]. In these Japanese studies, the use of MTX led to reduced pre-engraftment immune reactions, engraftment syndromes, acute GVHD, and TRM while improving RFS in both children and adults. Since Koreans are ethnically similar to Japanese, the use of MTX indeed could be beneficial but the number of patients in the present study is too small and the administration schedule too heterogeneous to firmly determine the benefits of MTX addition. On the other hand, the use of ATG warrants more caution. Although there are no large comparative studies, since CBT is a naturally T-cell depleted HSCT, the use of ATG during CBT has been traditionally associated prolonged T lymphopenia and subsequently higher infection rates [13,14,26]. In our study, there was no difference in infection rates between ATG-users versus non-users but the use of ATG was identified as an adverse prognostic factor for survival (Table 3), advocating avoidance of its use during CBT. 

For ALL, the results were a bit different. The RFS, OS, and cumulative incidence of acute GVHD in our ALL patients were similar to results of previous reports [27,28,29]. In our cohort, MAC conditioning was associated with trends towards better survival outcomes (Figure 2) and TBI-based MAC conditioning seemed especially beneficial. To the best of our knowledge, this is the first report showing the superiority of TBI-based MAC conditioning in CBT setting for adult ALL. This comes as no surprise, however, as TBI-based MAC conditioning has been consistently associated with better survival outcomes in ALL [30,31,32]. More meticulously executed prospective trials should ensue for confirmation of this particular finding. As for GVHD prophylaxis, the used of ATG was associated with negative survival outcomes in ALL as in AML. 

The advantage of single versus double CBT in adults is theoretical. Double CBT has shown improved 2-year survival with double CBT (62%) compared to single CBT (47%) in some reports [33], but a randomized study failed to prove the superiority [21]. Rather, the extent of HLA match and CB TNC dose seem to navigate the success of CBT [34]. In our study, most patients underwent double CBT to meet the recommended minimum number of TNC (3.0x10^7^/kg), but there were no survival differences between single CBT group versus double CBT group. 

**Figure 4:**
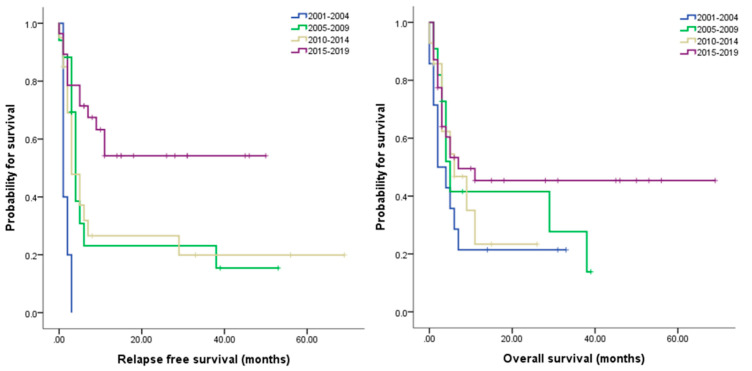
Relapse free survival (**left**) and overall survival (**right**) over time.

One of the most obvious limitations of this study is the retrospective nature. Furthermore, although 70 cases of CBT is not a trivial number considering the fact that the use of CBT is not as widespread, it is perhaps a little small to draw statistically powerful conclusions. However, the advantage of using Korean population is that because Korea has a single public medical insurance system that covers approximately 98% of the overall Korean population [35]. The range of coverage is strictly controlled; thus, the general leukemia treatment algorithm is relatively uniform throughout the population. This, in turn, ensures the quality of data. Lastly, since the duration of the study period spans from year 2000 to 2019, many aspects of acute leukemia diagnosis and treatment, including risk stratification, use of target therapies, supportive care, have altered over the course of time. However, if anything, from the results of CBT are improving as seen in Figure 4, we can safely assume that such changes had positive effects. All in all, these limitations do not diminish the importance of our findings that can be readily incorporated into real-world practice.

## 5. Conclusions 

In conclusion, we provide evidence that CBT is a readily available option worth considering for adult acute leukemia patients. The results of CBT are improving, but there is still room for improvement with regards to patient selection and optimal preparative regimen. Individualized conditioning regimens will add value in terms of enhancing safety and efficacy, and in the absence of established guidelines, this study provides aid for physicians in selecting appropriate preparative regimens.

## Figures and Tables

**Table 1:** Baseline characteristics.

	All *N* = 70 (%)	AML *N* = 41 (%)	ALL *N* = 29 (%)	*P*
**Age at transplantation, years (median, range)**	44 (18–66)	47 (22–66)	29 (18–64)	<0.001
**Sex, male**	35 (50.0)	18 (43.9)	17 (58.6)	0.225
**Body weight, kg (median, range)**	59.5 (43–98)	60 (43–90)	58 (45–98)	0.787
**Diagnosis to transplantation, months (median, range)**	7 (2–85)	9 (3–85)	7 (2–76)	0.425
**Transplantation period**				
2001–2004	5 (7.1)	4 (9.8)	1 (3.4)	0.542
2005–2009	17 (24.3)	9 (22.0)	8 (27.6)	
2010–2014	20 (28.6)	10 (24.4)	10 (34.5)	
2015–2019	28 (40.0)	18 (43.9)	10 (34.5)	
**Disease status at transplantation**				
CR1	43 (61.4)	24 (58.5)	19 (65.5)	0.195
CR2	13 (18.6)	6 (14.6)	7 (24.1)	
Others*	14 (20.0)	11 (26.8)	3 (10.3)	
**Conditioning regimen**				
Myeloablative				
TBI containing	31 (44.3)	16 (39.0)	15 (51.7)	0.608
Non-TBI containing	14 (20.0)	9 (22.0)	5 (17.2)	
Reduced intensity				
TBI containing	11 (15.7)	6 (14.6)	5 (17.2)	0.600
Non-TBI containing	14 (20.0)	10 (24.4)	4 (13.8)	
**GVHD prophylaxis**				
CNI + ATG	11 (15.7)	7 (17.1)	4 (13.8)	0.804
CNI + MTX	20 (28.6)	10 (24.4)	10 (34.5)	
CNI + MTX + ATG	9 (12.9)	6 (14.6)	3 (10.3)	
CNI only	30 (42.9)	18 (43.9)	12 (41.4)	
**Infused cord blood**				
Total nucleated cells, 10^7^/kg (median, range)	2.5 (1.0–5.2)	2.4 (1.0–5.2)	2.5 (1.0–4.1)	0.947
CD34+ cells, 10^5^/kg (median, range)	1.0 (0.1–4.1)	1.2 (0.1–4.1)	1.0 (0.1–2.7)	0.413
**Single unit HLA matching (*N* = 13)**				
6/6	1 (7.7)	1 (10.0)	0	0.557
5/6	2 (15.4)	1 (10.0)	1 (33.3)	
4/6	10 (76.9)	8 (80.0)	2 (66.7)	
**Double unit HLA matching (*N* = 57)**				
4/6 + 4/6	8 (14.0)	6 (19.4)	2 (7.7)	0.443
5/6 + 5/6	18 (31.6)	12 (38.7)	6 (23.1)	
6/6 + 6/6	5 (8.8)	2 (6.5)	3 (11.5)	
6/6 + 5/6	2 (3.5)	0	2 (7.7)	
6/6 + 4/6	2 (3.5)	1 (3.2)	1 (3.8)	
6/6 + 3/6	1 (1.8)	0	1 (3.8)	
5/6 + 4/6	19 (33.3)	9 (29.0)	10 (38.5)	
5/6 + 3/6	2 (3.5)	1 (3.8)	1 (3.8)	
**ECOG performance status at transplantation**				
0	22 (31.4)	13 (31.7)	9 (31.0)	0.747
1	41 (58.6)	23 (56.1)	18 (62.1)	
2	7 (10.0)	5 (12.2)	2 (6.9)	

AML: acute myeloid leukemia, ALL: acute lymphoblastic leukemia, CR1: first complete remission, CR2: a complete remission, TBI: total body irradiation, GVHD: graft-versus-host disease, CNI: calcineurin inhibitor, ATG: anti-thymoglobulin, MTX: methotrexate, HLA: human leukocyte antigen, ECOG: Eastern Cooperative Oncology Group. Others include CR beyond CR2 (CR3, CR4 and so forth) and salvage HSCT in refractory settings.

**Table 2:** Transplantation outcomes.

	All *N* = 70 (%)	AML *N* = 41 (%)	ALL *N* = 29 (%)
**Neutrophil engraftment**	55 (78.6)	32 (78.0)	23 (79.3)
Time to neutrophil engraftment, days (median, range)	22 (8–74)	21 (8–64)	26 (11–74)
**Platelet recovery**	46 (65.7)	27 (65.9)	19 (65.5)
Time to platelet recovery, days (median, range)	41 (15–182)	39 (15–182)	46 (20–131)
Cumulative incidence of any acute GVHD at Day 100 *	33.5	29.3	39.9
Grade II-IV acute GVHD	24.7	26.8	21.7
Cumulative incidence of any chronic GVHD at 1 year *	17.8	13.5	24.5
Moderate-severe chronic GVHD	3.2	2.5	4.4
**Any infection**	45 (64.3)	28 (68.3)	17 (58.6)
Bacterial ^†^	34 (75.6)	19 (70.4)	15 (83.3)
Fungal ^†^	6 (13.3)	5 (18.5)	1 (5.6)
Viral ^†^	5 (11.1)	3 (11.1)	2 (11.1)
**CMV antigenemia**	45 (65.2)	31 (75.6)	14 (48.3)
Pneumonitis	5	4	1
Retinitis	4	4	0
GI tract infection	5	4	1
Cumulative incidence of TRM at Day 100 *	29.2	29.3	28.9
Cumulative incidence of TRM at 1 year *	36.9	39.5	32.7

AML: acute myeloid leukemia, ALL: acute lymphoblastic leukemia, GVHD: graft-versus-host disease, CMV: cytomegalovirus, GI: gastrointestinal, TRM: treatment-related mortality. * Represented as % (95% confidence interval range) ^†^ Percentage out of all infections.

**Table 3:** Risk factors for transplantation outcomes on multivariate analyses.

	Variable		HR (95% CI)	*p*
**AML, relapse free survival**	Year of CBT	2001–2004	1	
		2005–2009	0.304 (0.084–1.099)	0.069
		2010–2014	0.285 (0.078–1.044)	0.058
		2015–2019	0.147 (0.040–0.535)	0.004
	GVHD prophylaxis	CNI + ATG	1	
		CNI + MTX	0.260 (0.070–0.968)	0.045
		CNI + ATG + MTX	1.147 (0.349–3.773)	0.822
		CNI only	0.647 (0.240–1.747)	0.391
**AML, overall survival**	Year of CBT	2001–2004	1	
		2005–2009	0.325 (0.090–1.168)	0.085
		2010–2014	0.226 (0.058–0.874)	0.031
		2015–2019	0.151 (0.041–0.556)	0.004
**ALL, relapse free survival**	Conditioning regimen	Group 1	1	
		Group 2	0.292 (0.053–1.604)	0.157
		Group 3	0.364 (0.072–1.841)	0.222
		Group 4	0.150 (0.033–0.678)	0.014
	Year of CBT	2001–2004	1	
		2005–2009	0.048 (0.003–0.853)	0.039
		2010–2014	0.062 (0.004–1.031)	0.053
		2015–2019	0.012 (0.001–0.267)	0.005
	GVHD prophylaxis	CNI + ATG	1	
		CNI + MTX	0.290 (0.052–1.604)	0.156
		CNI + ATG + MTX	1.107 (0.173–7.068)	0.914
		CNI only	0.150 (0.026–0.873)	0.035
**ALL, overall survival**	Conditioning regimen	Group 1	1	
		Group 2	0.183 (0.027–1.243)	0.082
		Group 3	0.398 (0.076–2.069)	0.273
		Group 4	0.189 (0.042–0.857)	0.031

AML: acute myeloid leukemia, CR1: first complete remission, CR2: a complete remission, CBT: cord blood transplantation, GVHD: graft-versus-host disease, CNI: calcineurin inhibitor, ATG.

## References

[1] Schoemans H., Theunissen K., Maertens J., Boogaerts M., Verfaillie C.M., Wagner J. (2006). Adult umbilical cord blood transplantation: A comprehensive review. Bone Marrow Transplant..

[2] Brown J.A., Boussiotis V.A. (2008). Umbilical cord blood transplantation: Basic biology and clinical challenges to immune reconstitution. Clin. Immunol..

[3] Robin M., Ruggeri A., Labopin M., Niederwieser D., Tabrizi R., Sanz G., Bourhis J.-H., Van Biezen A., Koenecke C., Blaise D. (2015). Comparison of Unrelated Cord Blood and Peripheral Blood Stem Cell Transplantation in Adults with Myelodysplastic Syndrome after Reduced-Intensity Conditioning Regimen: A Collaborative Study from Eurocord (Cord blood Committee of Cellular Therapy & Immunobiology Working Party of EBMT) and Chronic Malignancies Working Party. Biol. Blood Marrow Transplant..

[4] Atsuta Y., Suzuki R., Nagamura-Inoue T., Taniguchi S., Takahashi S., Kai S., Sakamaki H., Kouzai Y., Kasai M., Fukuda T. (2009). Disease-specific analyses of unrelated cord blood transplantation compared with unrelated bone marrow transplantation in adult patients with acute leukemia. Blood.

[5] Milano F., Gooley T., Wood B., Woolfrey A., Flowers M.E., Doney K., Witherspoon R., Mielcarek M., Deeg J.H., Sorror M. (2016). Cord-Blood Transplantation in Patients with Minimal Residual Disease. N. Engl. J. Med..

[6] Eapen M., Rocha V., Sanz G., Scaradavou A., Zhang M.-J., Arcese W., Sirvent A., Champlin R.E., Chao N., Gee A.P. (2010). Effect of graft source on unrelated donor haemopoietic stem-cell transplantation in adults with acute leukaemia: A retrospective analysis. Lancet Oncol..

[7] Eapen M., Rubinstein P., Zhang M.-J., Stevens C., Kurtzberg J., Scaradavou A., Loberiza F.R., E Champlin R., Klein J.P., Horowitz M.M. (2007). Outcomes of transplantation of unrelated donor umbilical cord blood and bone marrow in children with acute leukaemia: A comparison study. Lancet.

[8] Atsuta Y., Morishima Y., Suzuki R., Nagamura-Inoue T., Taniguchi S., Takahashi S., Kai S., Sakamaki H., Kouzai Y., Kobayashi N. (2012). Comparison of Unrelated Cord Blood Transplantation and HLA-Mismatched Unrelated Bone Marrow Transplantation for Adults with Leukemia. Biol. Blood Marrow Transplant..

[9] Rocha V., Labopin M., Sanz G., Arcese W., Schwerdtfeger R., Bosi A., Jacobsen N., Ruutu T., De Lima M., Finke J. (2004). Transplants of Umbilical-Cord Blood or Bone Marrow from Unrelated Donors in Adults with Acute Leukemia. N. Engl. J. Med..

[10] Takahashi S., Ooi J., Tomonari A., Konuma T., Tsukada N., Oiwa-Monna M., Fukuno K., Uchiyama M., Takasugi K., Iseki T. (2006). Comparative single-institute analysis of cord blood transplantation from unrelated donors with bone marrow or peripheral blood stem-cell transplants from related donors in adult patients with hematologic malignancies after myeloablative conditioning regimen. Blood.

[11] Glucksberg H., Storb R., Fefer A., Buckner C.D., Neiman P.E., Clift R.A., Lerner K.G., Thomas E.D. (1974). Clinical manifestations of graft-versus-host disease in human recipients of marrow from HL-A-matched sibling donors. Transplantation.

[12] Jagasia M.H., Greinix H.T., Arora M., Williams K.M., Wolff D., Cowen E.W., Palmer J., Weisdorf D., Treister N.S., Cheng G. (2015). National Institutes of Health Consensus Development Project on Criteria for Clinical Trials in Chronic Graft-versus-Host Disease: I. The 2014 Diagnosis and Staging Working Group report. Biol. Blood Marrow Transplant..

[13] Pascal L., Tucunduva L., Ruggeri A., Blaise D., Ceballos P., Chevallier P., Cornelissen J., Maillard N., Tabrizi R., Petersen E. (2015). Impact of ATG-containing reduced-intensity conditioning after single- or double-unit allogeneic cord blood transplantation. Blood.

[14] Pascal L., Mohty M., Ruggeri A., Tucunduva L., Milpied N., Chevallier P., Tabrizi R., Labalette M., Gluckman E., Labopin M. (2014). Impact of rabbit ATG-containing myeloablative conditioning regimens on the outcome of patients undergoing unrelated single-unit cord blood transplantation for hematological malignancies. Bone Marrow Transplant..

[15] Barker J.N., Weisdorf D.J., DeFor T., Blazar B.R., Miller J.S., E Wagner J. (2003). Rapid and complete donor chimerism in adult recipients of unrelated donor umbilical cord blood transplantation after reduced-intensity conditioning. Blood.

[16] Ballen K.K., Spitzer T.R., Yeap B.Y., McAfee S., Dey B.R., Attar E., Haspel R., Kao G., Liney D., Alyea E. (2007). Double Unrelated Reduced-Intensity Umbilical Cord Blood Transplantation in Adults. Biol. Blood Marrow Transplant..

[17] Brunstein C.G., Gutman J.A., Weisdorf D.J., Woolfrey A.E., DeFor T.E., Gooley T.A., Verneris M.R., Appelbaum F.R., Wagner J.E., Delaney C. (2010). Allogeneic hematopoietic cell transplantation for hematologic malignancy: Relative risks and benefits of double umbilical cord blood. Blood.

[18] Weisdorf D., Eapen M., Ruggeri A., Zhang M.-J., Zhong X., Brunstein C., Ustun C., Rocha V., Gluckman E. (2014). Alternative donor transplantation for older patients with acute myeloid leukemia in first complete remission: A center for international blood and marrow transplant research-eurocord analysis. Biol. Blood Marrow Transplant..

[19] De Latour R.P., Brunstein C.G., Porcher R., Chevallier P., Robin M., Warlick E., Xhaard A., Ustun C., Larghéro J., Dhédin N. (2013). Similar Overall Survival Using Sibling, Unrelated Donor, and Cord Blood Grafts after Reduced-Intensity Conditioning for Older Patients with Acute Myelogenous Leukemia. Biol. Blood Marrow Transplant..

[20] Brunstein C.G., Fuchs E.J., Carter S.L., Karanes C., Costa L.J., Wu J., Devine S.M., Wingard J.R., Aljitawi O.S., Cutler C.S. (2011). Alternative donor transplantation after reduced intensity conditioning: Results of parallel phase 2 trials using partially HLA-mismatched related bone marrow or unrelated double umbilical cord blood grafts. Blood.

[21] Ballen K.K., Lazarus H. (2016). Cord blood transplant for acute myeloid leukaemia. Br. J. Haematol..

[22] Narimatsu H., Terakura S., Matsuo K., Oba T., Uchida T., Iida H., Hamaguchi M., Watanabe M., Kohno A., Murata M. (2006). Short-term methotrexate could reduce early immune reactions and improve outcomes in umbilical cord blood transplantation for adults. Bone Marrow Transplant..

[23] Terakura S., Azuma E., Murata M., Kumamoto T., Hirayama M., Atsuta Y., Kodera Y., Yazaki M., Naoe T., Kato K. (2007). Hematopoietic Engraftment in Recipients of Unrelated Donor Umbilical Cord Blood Is Affected by the CD34+ and CD8+ Cell Doses. Biol. Blood Marrow Transplant..

[24] Takahashi S., Iseki T., Ooi J., Tomonari A., Takasugi K., Shimohakamada Y., Yamada T., Uchimaru K., Tojo A., Shirafuji N. (2004). Single-institute comparative analysis of unrelated bone marrow transplantation and cord blood transplantation for adult patients with hematologic malignancies. Blood.

[25] Kato K., Yoshimi A., Ito E., Oki K., Hara J., Nagatoshi Y., Kikuchi A., Kobayashi R., Nagamura-Inoue T., Kai S. (2011). Cord Blood Transplantation from Unrelated Donors for Children with Acute Lymphoblastic Leukemia in Japan: The Impact of Methotrexate on Clinical Outcomes. Biol. Blood Marrow Transplant..

[26] Komanduri K.V., John L.S.S., De Lima M., McMannis J., Rosinski S., Mcniece I., Bryan S.G., Kaur I., Martin S., Wieder E.D. (2007). Delayed immune reconstitution after cord blood transplantation is characterized by impaired thymopoiesis and late memory T-cell skewing. Blood.

[27] Matsumura T., Network F.T.J.C.B.B., Kami M., Yamaguchi T., Yuji K., Kusumi E., Taniguchi S., Takahashi S., Okada M., Sakamaki H. (2012). Allogeneic cord blood transplantation for adult acute lymphoblastic leukemia: Retrospective survey involving 256 patients in Japan. Leukemia.

[28] I Marks D., Aversa F., Lazarus H.M. (2006). Alternative donor transplants for adult acute lymphoblastic leukaemia: A comparison of the three major options. Bone Marrow Transplant..

[29] Marks D.I., Woo K.A., Zhong X., Appelbaum F.R., Bachanova V., Barker J.N., Brunstein C.G., Gibson J., Kebriaei P., Lazarus H.M. (2013). Unrelated umbilical cord blood transplant for adult acute lymphoblastic leukemia in first and second complete remission: A comparison with allografts from adult unrelated donors. Haematologica.

[30] Davies S.M., Ramsay N.K.C., Klein J.P., Weisdorf D.J., Bolwell B., Cahn J.-Y., Camitta B.M., Gale R.P., Giralt S., Heilmann C. (2000). Comparison of Preparative Regimens in Transplants for Children With Acute Lymphoblastic Leukemia. J. Clin. Oncol..

[31] Bunin N.J., Aplenc R., Kamani N., Shaw K., Cnaan A., Simms S. (2003). Randomized trial of busulfan vs total body irradiation containing conditioning regimens for children with acute lymphoblastic leukemia: A Pediatric Blood and Marrow Transplant Consortium study. Bone Marrow Transplant..

[32] Cahu X., Ebmt O.B.O.T.A.L.W.P.O., Labopin M., Giebel S., Aljurf M., Kyrcz-Krzemien S., Socie G., Eder M., Bonifazi F., Bunjes D. (2015). Impact of conditioning with TBI in adult patients with T-cell ALL who receive a myeloablative allogeneic stem cell transplantation: A report from the acute leukemia working party of EBMT. Bone Marrow Transplant..

[33] Labopin M., Ruggeri A., Gorin N.C., Gluckman E., Blaise D., Mannone L., Milpied N., Yakoub-Agha I., Deconinck E., Michallet M. (2013). Cost-effectiveness and clinical outcomes of double versus single cord blood transplantation in adults with acute leukemia in France. Haematologica.

[34] Barker J.N., Scaradavou A., Stevens C.E. (2010). Combined effect of total nucleated cell dose and HLA match on transplantation outcome in 1061 cord blood recipients with hematologic malignancies. Blood.

[35] Kim D.S. (2010). Introduction: Health of the Health Care System in Korea. Soc. Work. Public Health.

